# Magnetic resonance imaging to assess the brain response to fasting in glioblastoma-bearing rats as a model of cancer anorexia

**DOI:** 10.1186/s40644-023-00553-y

**Published:** 2023-04-10

**Authors:** Irene Guadilla, Sara González, Sebastián Cerdán, Blanca Lizarbe, Pilar López-Larrubia

**Affiliations:** 1 Biomedical Magnetic Resonance Group, Instituto de Investigaciones Biomédicas Alberto Sols, CSIC-UAM, C/ Arturo Duperier 4, 28029 Madrid, Spain; 2 Departamento de Bioquímica, Universidad Autónoma de Madrid, 28029 Madrid, Spain

**Keywords:** Diffusion tensor imaging, Manganese enhanced MRI, Glioblastoma, Cancer anorexia, Fasting paradigm

## Abstract

**Background:**

Global energy balance is a vital process tightly regulated by the brain that frequently becomes dysregulated during the development of cancer. Glioblastoma (GBM) is one of the most investigated malignancies, but its appetite-related disorders, like anorexia/cachexia symptoms, remain poorly understood.

**Methods:**

We performed manganese enhanced magnetic resonance imaging (MEMRI) and subsequent diffusion tensor imaging (DTI), in adult male GBM-bearing (*n* = 13) or control Wistar rats (*n* = 12). A generalized linear model approach was used to assess the effects of fasting in different brain regions involved in the regulation of the global energy metabolism: cortex, hippocampus, hypothalamus and thalamus. The regions were selected on the contralateral side in tumor-bearing animals, and on the left hemisphere in control rats. An additional DTI-only experiment was completed in two additional GBM (*n* = 5) or healthy cohorts (*n* = 6) to assess the effects of manganese infusion on diffusion measurements.

**Results:**

MEMRI results showed lower T_1_ values in the cortex (*p*-value < 0.001) and thalamus (*p*-value < 0.05) of the fed ad libitum GBM animals, as compared to the control cohort, consistent with increased Mn^2+^ accumulation. No MEMRI-detectable differences were reported between fed or fasting rats, either in control or in the GBM group. In the MnCl_2_-infused cohorts, DTI studies showed no mean diffusivity (MD) variations from the fed to the fasted state in any animal cohort. However, the DTI-only set of acquisitions yielded remarkably decreased MD values after fasting only in the healthy control rats (*p*-value < 0.001), and in all regions, but thalamus, of GBM compared to control animals in the fed state (*p*-value < 0.01). Fractional anisotropy (FA) decreased in tumor-bearing rats due to the infiltrate nature of the tumor, which was detected in both diffusion sets, with (*p*-value < 0.01) and without Mn^2+^ administration (*p*-value < 0.001).

**Conclusions:**

Our results revealed that an altered physiological brain response to fasting occurred in hunger related regions in GBM animals, detectable with DTI, but not with MEMRI acquisitions. Furthermore, the present results showed that Mn^2+^ induces neurotoxic inflammation, which interferes with diffusion MRI to detect appetite-induced responses through MD changes.

**Supplementary Information:**

The online version contains supplementary material available at 10.1186/s40644-023-00553-y.

## Background

Global energy balance is a complex systemic response that involves the coordination of numerous organs, including the brain. A plethora of dedicated neuronal pathways located in different cerebral regions coordinates the homeostatic adjustment between food intake and energy expenditure [1]. This delicate homeostatic equilibrium is found to be perturbed in many neurological disorders, such as in patients with malignant tumors, who usually develop anorexia (loss of appetite), a major component of cancer cachexia [2]. In fact, anorexia/cachexia syndrome is one of the most common causes of death among cancer patients and, in advanced stages, can reach approximately 80% of cases [3]. Although malignant gliomas are among the most investigated tumors, the mechanisms underlying their devastating appetite-related consequences remain poorly understood. Indeed, although anorexia and body weight loss are specific signs of cachexia in glioma patients, the symptoms of glioma associated with cachexia have received little attention [4, 5]. It is also important to note that fatigue, as another important symptom of cachexia, has a prevalence in glioma patients ranging from 39 to 77% [6].

Magnetic resonance imaging (MRI) is used routinely to obtain morphological and functional information of the brain. In particular, diffusion weighted imaging (DWI) and diffusion tensor imaging (DTI) can characterize cerebral microstructure [7, 8]. Their usage already extends to a wide range of cerebral pathologies, including oncological diseases, ischemic episodes, neurodegeneration, mental disorders and brain trauma [9, 10]. Complementary information on cerebral activation may be gathered from functional neuroimaging perspectives [11], including diffusion functional MRI [12], or manganese enhanced magnetic resonance imaging (MEMRI) [13].

Briefly, diffusion functional studies are based on MRI detection of changes in translational motion of water molecules resulting from activation-induced ionic transferring and associated neurocellular swelling/shrinking events. Such effects can be evaluated by quantifying the alterations in the diffusion coefficients, like mean diffusivity (MD) and fractional anisotropy (FA), using mono-exponential [14, 15], bi-exponential, or even tri-exponential models that can separate the diffusion regimes of water molecules into different compartments [16, 17]. Previous research demonstrated that diffusion MRI protocols are able to detect fasting-induced alterations in the brains of mice, rats and humans [18], suggesting that they could provide valuable information about the effects of tumor growth on brain regulation of global energy homeostasis.

MEMRI, on the other hand, can be used as a preclinical functional imaging methodology in animal models and it is based on the systemic administration of a manganese ion (Mn^2+^) solution to reveal areas of neural activity. This is achieved by tracking the Mn^2+^ accumulation in neural cells during neurotransmission, which occurs by its competition with the calcium ion (Ca^2+^) in voltage-gated Ca^2+^ channels [19, 20]. Mn^2+^ is a paramagnetic cation that induces spin–lattice relaxation time (T_1_) shortening proportional to its concentration accumulation, thus revealing areas of neuronal activation with brighter enhancement in T_1_ weighted (T_1_W) images [21]. Mn^2+^ accumulates in the neuronal endoplasmic reticulum, is transported through axonal tracts and reaches the presynaptic membrane. It is then released into the synaptic cleft and finally uptaken by the postsynaptic neuron [22]. This fact allows the generation of functional maps of neural pathways acting as a trans-synaptic, tract-tracing methodology, identifying perturbations that can be associated with neuropathologies [23]. In the rodent brain, studies using MEMRI with systemic infusions of MnCl_2_ detected altered hypothalamic neuronal activity associated with fasting [24, 25], and the corresponding effects in genetically obese mice [26]. However, the use of MEMRI is limited to preclinical studies due to its associated neurotoxicity [27]. Notably, although the potential of MEMRI and DTI in the assessment of functional anatomy has been explored [28, 29], the compatibility of both methodologies in the evaluation of brain responses to a stimulus have not yet been investigated to our knowledge.

We hypothesized that brain activity underlying physiological responses to hunger might be disrupted or suppressed in glioblastoma (GBM) bearing animals and be detected by DTI and MEMRI methodologies. This must probably occur in specific brain areas involved in the regulation of food intake, such as the cortex, hippocampus, hypothalamus and thalamus [30]. The abnormal imaging findings at specific hunger-related regions in GBM-bearing animals may represent underlying alterations in neuronal activity and suggest the contribution of GBM to cachexia in GBM patients. The ability to assess it could make an important contribution to the monitoring of therapies aimed at improving appetite disorders in cancer patients.

## Materials and methods

### Animals and experimental design

All experimental procedures were approved by the Ethic Committees of the Biomedical Research Institute “Alberto Sols”, the Spanish National Research Council (CSIC) and the Community of Madrid (PROEX 047/18) and follow the national (R.D.53/2013) and European Community guidelines (2010/62/UE) for care and management of experimental animals. The design and reporting adhered to the ARRIVE guidelines [31]. Rats were housed in the animal premises of our institution (Reg. No. ES280790000188) and cared for by specialized personnel.

Male adult Wistar rats (own production) (*n* = 25, 220–250 g, body weight, b.w.) were randomly separated in two main groups: control (*n* = 12) and tumor-bearing animals (*n* = 13). For the GBM model group, rat glioma C6 cells (ATCC® CCL-107 TM) were used after growing in Dulbecco's Modified Eagle Medium (DMEM) modified with 10% FBS, streptomycin sulfate (0.12 mL/400 mL of DMEM), and bencilpeniciline (0.14 mL/400 mL of DMEM). Rats were given inhalatory anesthesia inside a box (2–2.5% isoflurane/oxygen) and then placed on a stereotaxic device (Model 900LS Small Animal Stereotaxic Instrument, Kopf Instruments®) where 10^5^ cells in 10 μl of DMEM medium were injected in the right caudate nucleus using bregma as skull landmark: 0.35 mm right-lateral and 0.55 mm ventral [32]. Animals received buprenorphine (0.03 mg/kg, b.w.) subcutaneously as analgesic right after the surgery and the following two days. Tumor growth was followed with anatomical spin–spin relaxation time weighted (T_2_W) MR images, acquired every 3 days, starting 10 days after the intracranial injection of the glioma cells. When the gliomas were in an advanced stage, MRI studies were performed on the animals to assess brain response to food deprivation.

Control and GBM animals were subdivided into two groups to be assessed under fed ad libitum (6 control and 6 GBM) or fasting conditions after 16 h of food deprivation (6 control and 7 GBM) (Supplementary Fig. 1A). A solution of MnCl_2_ (100 mM, pH = 7.4) was injected subcutaneously by using an infusion pump and a cannula tubing placed in the abdominal area at a dosage of 1 mmol/kg b.w. and a flow rate of 0.1 mL/min. Mn^2+^ infusions were performed 24 h prior to MRI acquisitionsunder inhalational anesthesia [20].

Additional animals (*n* = 11, 220–250 g, b.w.) were randomly divided in two new cohorts: control (*n* = 6) and GBM (*n* = 5). They were used to evaluate DTI parameters under feeding and, 24 h later, under fasting conditions in the absence of Mn^2+^ solution administration (Supplementary Fig. 1B).

#### *Magnetic resonance imaging studies*

Image acquisitions were carried out in a Bruker BioSpec® 7 T system (Bruker Biospin, Ettlingen, DE), 16 cm bore, with a 90 mm gradient insert of 360 mT/m and a 40 mm quadrature volume resonator, interfaced with an Avance III radiofrequency console and running ParaVision 5.1 software.

Anesthetized animals (induced with 2–2.5% and maintained with 1–1.5% isoflurane/oxygen) were placed in a methyl methacrylate rat-holder of the MRI system and restrained with a bite-bar attached to a nose mask and a small piece of tape over the head and attached to the animal’s bedding. Anatomical images acquired to monitor tumor development consisted of a RARE fast spin-echo T_2_W acquisition, with the following parameters: repetition time (TR) = 3000 ms, echo time (TE) = 44 ms, averages (Av) = 2, RARE factor = 8, 10 continuous slices with 1.5 mm of thickness in axial orientation, matrix data (Mtx) = 256 × 256 and 0.14 × 0.14 mm^2^/pixel in plane resolution.

When the tumors reached an advanced stage (volume > 100 mm^3^, coincident with high state of physical deterioration), DTI and MEMRI evaluations were performed 24 h after Mn^2+^ infusion to achieve the greatest effect on T_1_W image signal intensity. Each day, two animals were studied at the same time interval. To minimize bias, animals were randomly selected independently of experimental cohort (GBM or control) and feeding status (fed or fasted). To avoid circadian variations in neuronal activity [33], the imaging sessions always began at 8 a.m.. Prior to the imaging session, all rats were weighed on an electronic balance (0.1 g accuracy), and blood glucose levels were measured with a standard glucometer from a drop of blood from the tail tip. Once in the rat holder, a circulating warm water blanket was placed under the animal to maintain a stable body temperature during imaging acquisition. A small animal monitoring device (Model 1025, SA Instruments, Inc. NY) was used to follow the physiological status of the rats by measuring rectal temperature with an electronic thermometer and respiratory rate with a pneumatic pillow placed in the abdominal region. The respiratory rate was maintained between 40 and 60 breaths per minute by regulating the concentration of the anesthetic administered (1–1.5% isoflurane/oxygen).

The MRI protocol designed to evaluate the brain response to hunger in rats included two sets of acquisitions. A non-gated DTI study was performed employing the following features and parameters: TR = 2500 ms, TE = 43 ms, Av = 5, 5 slices in axial orientation with slice thickness = 1.5 mm and no-gap, Mtx = 128 × 128, 0.25 × 0.25 mm^2^/pixel in-plane resolution, single-shot echo-planar imaging (ss-EPI), diffusion gradients applied in 6 non-coplanar and non-collinear directions, gradient diffusion separation (Δ) = 20 ms, gradient diffusion duration (δ) = 4 ms, and b values of 0, 200 and 1000 s/mm^2^. Then, the MEMRI evaluation was carried out by employing a saturation-recovery experiment with a RARE sequence at variable TR to generate T_1_ maps with the following parameters: 7 TRs (150, 200, 400, 800, 1600, 3500 and 6000 ms), TE = 12.6 ms, Av = 1, 5 slices in axial orientation with slice thickness = 1.5 mm and no-gap, Mtx = 128 × 128 and 0.25 × 0.25 mm^2^/pixel in-plane resolution. The total acquisition time of the study was 45 min for each animal in the MEMRI + DTI study and 20 min in rats in the DTI-only evaluation.

### Image processing and data analysis

Data from MRI studies were processed with MyMapAnalyzer, a homemade script developed in MATLAB (R2010b, the MathWorks Inc., Natick, MA) to generate the parametric images. MRI datasets from the DTI acquisitions were fit in a pixel-by-pixel base to a monoexponential model to generate mean diffusivity (MD) and fractional anisotropy (FA) maps [34].

MRI signals of T_1_W images were fitted in a pixel-by-pixel base to Eq. 1 to obtain the corresponding T_1_ maps.

1 $${S}_{(TR)}={S}_{0}(1-{e}^{(-TR/{T}_{1})})$$

where *S*_*(TR)*_ is the signal intensity at a specific TR value, *S*_*0*_ is the signal intensity at a hypothetical TR = 0, and TR are each one of the 7 TR values employed (from 150 to 6000 ms).

In all cases, images were computed without any additional pre-processing procedures. As a measure of the goodness-of-fit to the estimated linear (for T_1_ maps) and multiple (for DTI) regression equations, only pixels with a value of R^2^ > 0.75 were included in the subsequent analysis. Four regions of interest (ROIs) related to the brain’s response to hunger [35, 36] -cortex, hippocampus, hypothalamus and thalamus- were assessed on the contralateral side in tumor-bearing animals in order to avoid imaging effects related to the GBM itself [37]. The same four ROIs were defined in the left hemisphere in control rats. ROIs containing 20–22 pixels (approximately 2 mm^3^) were manually selected on T_2_W images with an anatomical rat brain atlas as a guide [38], and overlaid on the parametric maps. For the selection of the ROIs, the same central slice was always used, in which the four regions can be unequivocally identified. Finally, the parameters were measured with ImageJ software (National Institutes of Health, Bethesda, MD, USA, ImageJ) and subsequently individual data from all pixels of the ROIs were statistically evaluated. Image processing and analysis was performed blinded to fed/fasted conditions.

### Statistical analysis

Statistical analyses to assess body weight and blood glucose levels differences in the four groups (control fed, control fasted, tumor-bearing fed, tumor-bearing fasted) were performed by corresponding unpaired multiple t-tests (fed vs fasted and control vs tumor-bearing) corrected with the Holm-Sidak method using GraphPad Prism software (GraphPad Software, La Jolla, CA, USA). The MRI parametric data were statistically analyzed using the IBM SPSS package (IBM SPSS Statistics for Windows, Version 25.0, Armonk, NY, IBM Corp.). Comparison of ROI data derived from parametric maps of animals in different experimental conditions was performed using a generalized linear model (GzLM) with generalized estimating equations (GEE). In this model, the dependent variables (MD, FA and T_1_) were related to the independent factors (control or tumor) and condition (fed or fasted). Data are presented as mean ± one standard deviation. In all cases, only *p*-values smaller than 0.05 were considered to be statistically significant.

## Results

### Physiological results

**Fig. 1:**
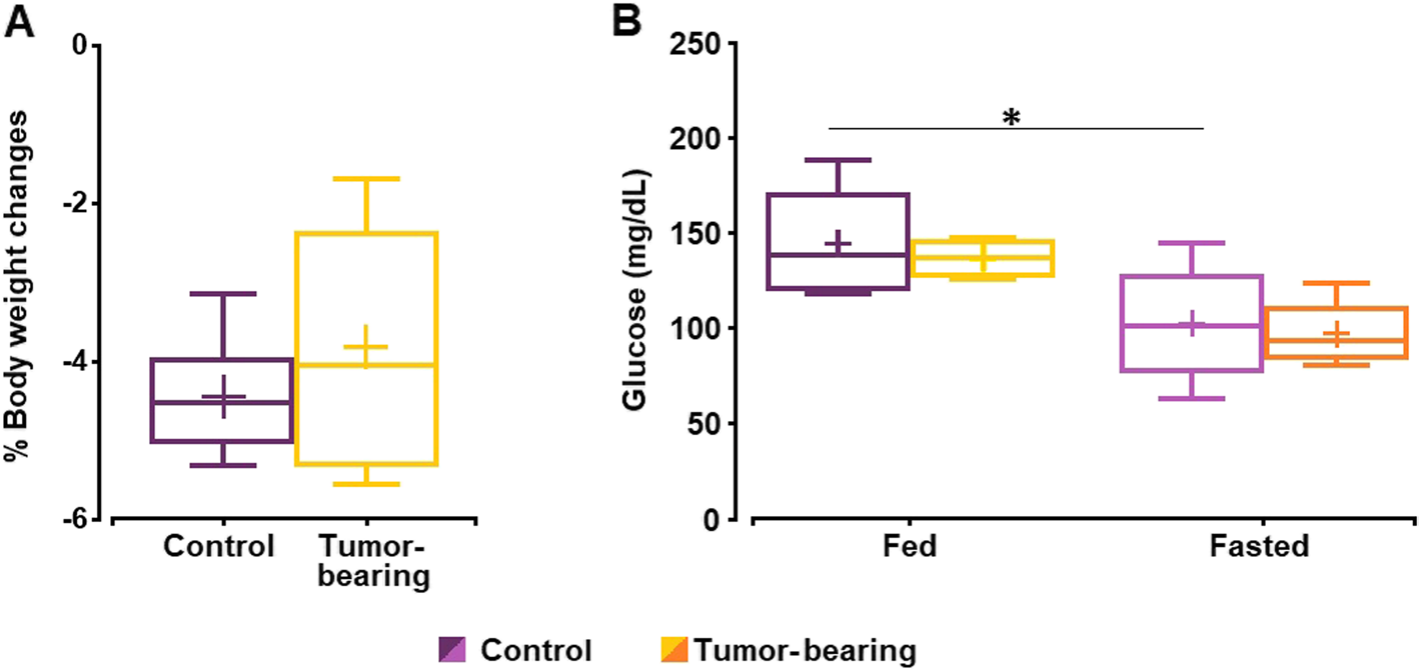
Decrease in body weight and blood glucose level in control and GBM rats under fed or fasted conditions. **A** Body weight changes after 16 h of food deprivation. **B** Blood glucose levels before the MRI session in fed and fasted conditions. In each box-plot, the central mark indicates the median, the cross mark the mean, and the bottom and top edges refer to the 25-th and 75-th percentiles, respectively. The upper and lower limits of the box extend to the most extreme data points not considered outliers (* *p*-value < 0.05)

Overnight fasting resulted in a significant decrease of body weight (measured 24 h apart) in healthy but not in tumor-bearing animals: control fed (241.7 ± 7.6 g) vs control fasted (230.9 ± 8.4 g) had a *p*-value = 0.05, while tumor fed (281.6 ± 30.0 g) vs tumor fasted (270.9 ± 30.2 g) had a *p*-value = 0.49. In any case, a similar percentage of weight lost was achieved for both control and GBM cohorts (Fig. 1A). Glucose concentration decreased with fasting in both groups, although it reached statistical significance in the control group (Fig. 1B).

### Effects of fasting in tumor-bearing animals assessed with MRI

**Fig. 2:**
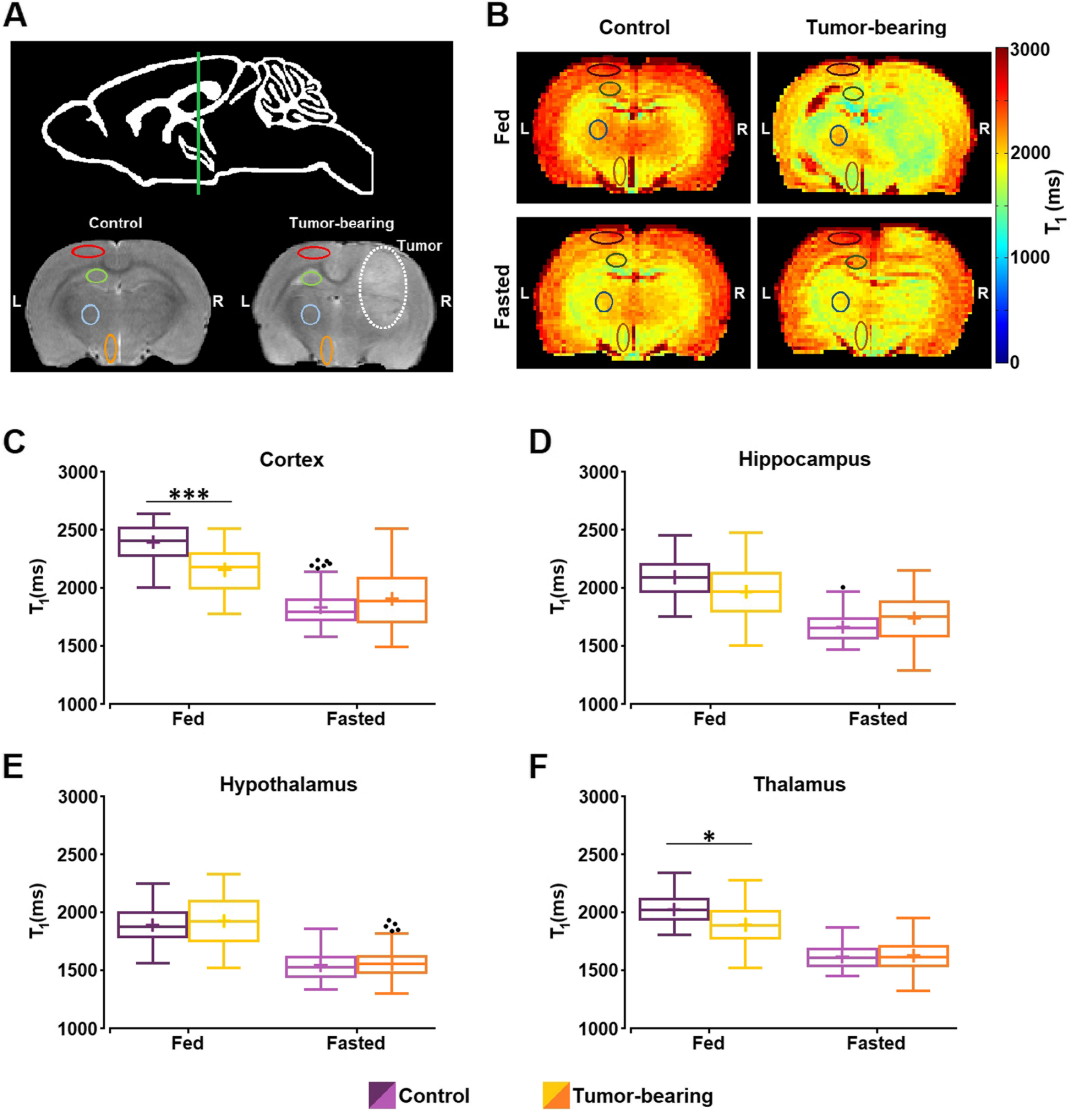
MEMRI of control and GBM rats, 24 h after Mn^2+^ administration, under fed or fasted conditions. **A** Anatomical location of the slice selected, including the perimeters of the investigated ROIs in the left contralateral hemisphere: cortex (red), hippocampus (green), thalamus (blue) and hypothalamus (orange). **B** Representative T_1_ maps of a control and a tumor-bearing animal, 24 h after Mn.^2+^ administration, under fed and fasted conditions. The ROIs (cortex, hippocampus, hypothalamus and thalamus) are outlined in the maps. **C-F** Boxplots of T_1_ values from the different ROIs investigated, in control and GBM rats, under fed and fasted conditions. Box plots are represented as indicated in Fig. 1. Outliers are plotted individually using the '•' symbol. (* *p*-value < 0.05, *** *p*-value < 0.001)

MEMRI and DTI studies were performed in control and tumor-bearing rats under both ad libitum feeding and fasting conditions. Representative images (T_2_W, T_1_W, and diffusion images) of a rat with GBM obtained in a MRI session are presented in Supplementary Fig. 2. The ROIs of the different regions investigated (Fig. 2A), were manually selected in T_2_W images and then exported to the corresponding parametric images.

Representative T_1_ maps of Mn^2+^-infused animals (Fig. 2B) for each state (control or tumor-bearing) and feeding condition (fed or 16 h-fasted), and the corresponding data are summarized in Fig. 2C-F and Supplementary Table 1, respectively. Statistical analysis of the data with GEE using feeding state, condition and region as independent factors revealed a significant effect of region (*p*-value < 0.001) and the product “state *x* condition *x* region” (*p*-value < 0.001). Pairwise analysis revealed that in the ad libitum feeding condition, control animals had significantly higher T_1_ values than tumor-bearing rats in both cortical (*p*-value = 0.001) and thalamic (*p*-value = 0.019) regions (Fig. 2C and F, respectively), but not in the hippocampus or hypothalamus (Fig. 2D and E, respectively). Comparison of T_1_ data for fasted animals did not reach statistical significance in any region. No significant differences were detected when comparing ROIs between feeding conditions in either control or GBM-bearing rats (Supplementary Table 1).

**Fig. 3:**
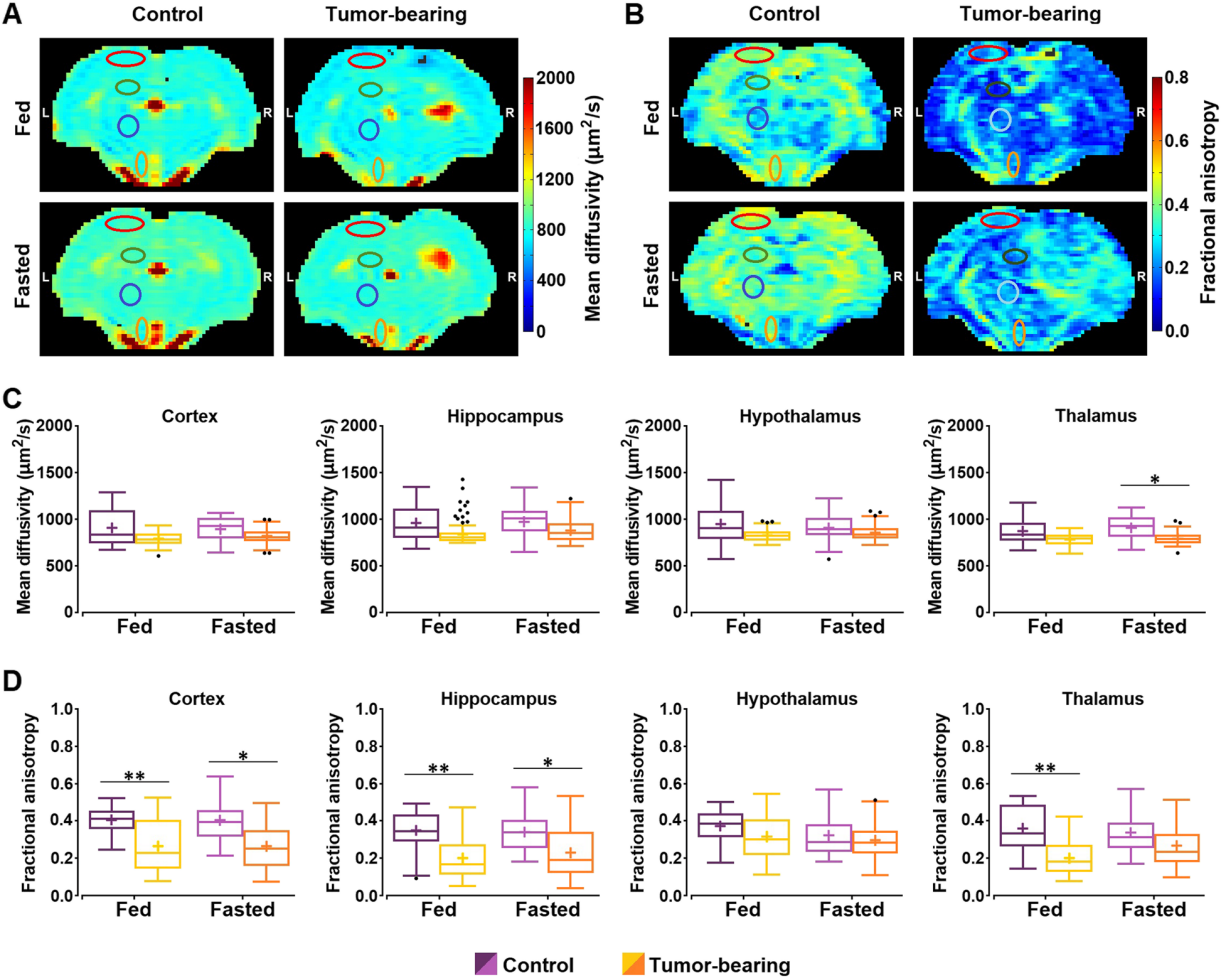
Mean diffusivity and fractional anisotropy in the brain of control and GBM rats under fed or fasted conditions 24 h after Mn^2+^ administration. **A**, **B** Representative parametric maps of mean diffusivity (MD) and fractional anisotropy (FA) of a control and a tumor-bearing rat, under fed and fasted conditions, respectively. The ROIs (cortex, hippocampus, hypothalamus and thalamus) are outlined in the maps. **C**, **D** Boxplots of the MD and FA values, respectively, from the different regions investigated (cortex, hippocampus, hypothalamus and thalamus) in control cohort and tumor-bearing rats under fed and fasted conditions. Box plots are represented as indicated in Fig. 1. Outliers are plotted individually using the '•' symbol. (* *p*-value < 0.05, ** *p*-value < 0.01)

The results of DTI studies performed during the MEMRI imaging session are presented in Fig. 3 with illustrative MD (Fig. 3A) and FA (Fig. 3B) maps of control and rats with GBM under both feeding and fasting conditions. The corresponding data are also shown in Supplementary Table 2. The GEE approach of the MD values obtained revealed a significant effect of the feeding state (*p*-value = 0.022), region (*p*-value < 0.001) and the product “state *x* condition *x* region” (*p*-value < 0.001). Pairwise comparisons of this parameter between controls and tumor-bearing animals in the fed measurements depicted no significant differences in any region. In the fasting condition, only the thalamus presented significantly lower MD values in rats with GBM compared to control animals (Fig. 3C). GEE assessment of the FA data revealed a significant effect of the state (*p*-value = 0.002), region (*p*-value < 0.001) and the product “state *x* condition *x* region” (*p*-value < 0.001). FA values were significantly lower in the cortex, hippocampus and thalamus of tumor-bearing rats in the fed condition compared to control (*p*-value < 0.01 in the three ROIs). Under fasting the cortex and hippocampus of GBM animals showed significantly lower values (*p*-value < 0.05 in the two ROIs) (Fig. 3D).

Finally, none of the DTI parameters present significant differences between feeding conditions in any region, when evaluated separately, either in the control or in the GBM cohorts (Supplementary Table 2).

**Fig. 4:**
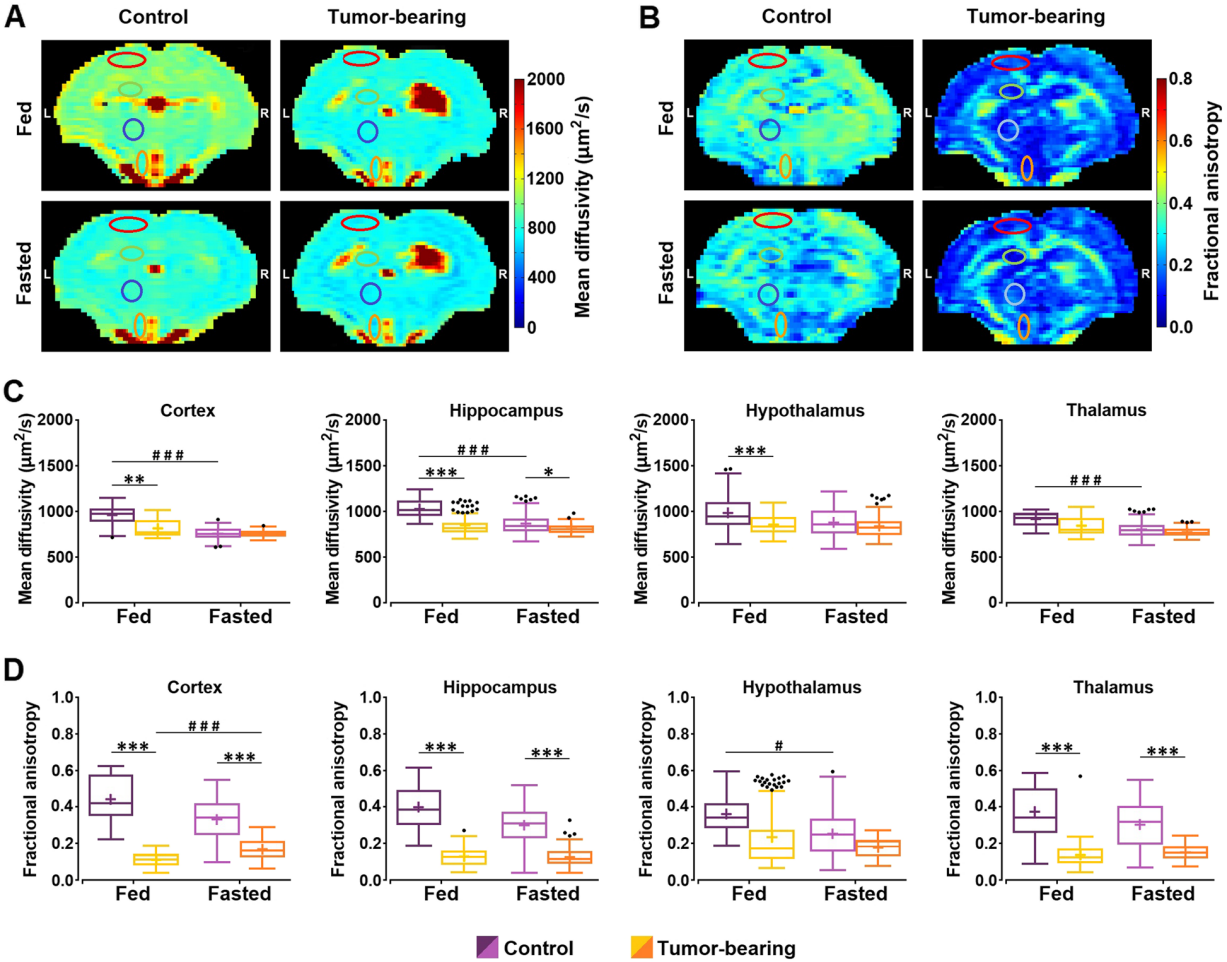
Mean diffusivity and fractional anisotropy in the brain of control and GBM rats under fed or fasted conditions without Mn.^2+^ administration. **A**, **B** Representative parametric maps of mean diffusivity (MD) and fractional anisotropy (FA) of a control and a tumor-bearing rat, under fed and fasted conditions, respectively. The ROIs (cortex, hippocampus, hypothalamus and thalamus) are outlined in the maps. **C**, **D** Boxplots of the MD and FA values, respectively, from the different regions investigated (cortex, hippocampus, hypothalamus and thalamus) in control cohort and tumor-bearing rats under fed and fasted conditions. Box plots are represented as indicated in Fig. 1. Outliers are plotted individually using the '•' symbol. (*/# *p*-value < 0.05, ** p-value < 0.01, ***/### *p*-value < 0.001)

To test whether the absence of DTI changes with fasting or with tumor presence was related to the MnCl_2_ infusion, DTI studies were acquired on a new set of animal cohorts (6 control and 5 GBM) under the same experimental protocol (fed ad libitum and after 16 h of food deprivation), without MnCl_2_ administration. Representative maps of non-Mn^2+^ infused animals are shown as examples for each parameter (MD and FA), pathological state (control and tumor) and feeding status (fed and fasted) (Fig. 4A and B). The corresponding MD and FA results are presented (Fig. 4C and D, respectively) and tabulated (Supplementary Table 3). Statistical evaluation of the MD values exposed the significant effect of state (*p*-value = 0.001), condition (*p*-value < 0.001), region (*p*-value < 0.001), and the product “state *x* condition *x* region” (*p*-value < 0.001). In the fed condition, MD in tumor-bearing rats was significantly lower than in control animals in the cortex, hippocampus and hypothalamus, while under fasting, significance was maintained only in hippocampus (Fig. 4C). Furthermore, food deprivation in control animals induced a decrease in MD, reaching statistical significance (*p*-value < 0.001) in the cortex, hippocampus and thalamus, but this event did not occur in GBM rats (Fig. 4C). The GEE approach also revealed that FA values were significantly affected by state, region and the product “state *x* condition *x* region” (*p*-value < 0.001 for all cases). Data showed significantly lower FA in the tumor group than in the control cohort in the cortex, hippocampus and thalamus, in both fed and fasting condition (*p*-value < 0.001) (Fig. 4D).

## Discussion

MRI techniques have evolved into very powerful tools to non-invasively characterize the anatomy and function of the normal brain and its pathologies. In particular, diffusion imaging and MEMRI methods allow for the assessment of physiological processes directly related to neural activation and cerebral microarchitecture. Previous research has used diffusion techniques or MEMRI to investigate brain mechanisms related to appetite and hunger [25, 35, 39], but to our knowledge, the effects of a brain tumor development on such feeding mechanisms had not previously been addressed. Moreover, previous works describe the use of MEMRI and DTI in vivo and ex vivo to evaluate the brain integrity, mainly the white matter, in different neuropathological conditions [40, 41]. Although previous work has used both MRI approaches in the same animals and imaging sessions to assess the axonal uptake and transport optic nerve [42], the use of both methodologies in the assessment of brain responses to feeding-related stimuli is lacking. In this work, we combine and compare DTI and MEMRI approaches to investigate brain responses to starvation in a rat model of glioblastoma by evaluating related imaging parameters in feeding and fasting states.

Different brain regions are involved in the regulation of the global energy metabolism [30, 43], several of which are investigated in this work. The hypothalamus is the most significant due to the high number of orexigenic and anorexigenic cells [44]. The cortex acts as the primary gustatory region that processes the odor and texture of food and perceived pleasantness. The hippocampus has a high concentration of receptors for ghrelin, insulin and leptin hormones which are related to appetite regulation [45]. The thalamus, in turn, receives inputs from appetite-related hypothalamic nuclei [36]. Thus, these regions were investigated in the in vivo MRI study presented here.

In our work, the physiological data showed that all animals experienced a decrease in body weight due to food deprivation with similar percentages of reduction in the control and GBM cohorts. Such weight loss, however, only reached statistical significance in the control animals. We suspect that the greater interindividual variability in the b.w. evolution of GBM-bearing animals after de C6 cells injection may be undermining the significance of this tendency. Similarly, all animals showed lower fasting glucose levels, but the difference was significant in control rats, suggesting an abnormal response to fasting in rats with glioma. Taken together, both results point to a potentially altered regulation of food intake and glycemic response to food deprivation on GBM rats, the latter deserving to be evaluated in future studies.

MEMRI methodologies have been widely used to map neuronal pathways, define morphological boundaries and study connectivity in structural and brain activation imaging studies [21, 46, 47]. Mn^2+^ enters and accumulates in excitable cells through voltage-gated Ca^2+^ channels. Since the increase in T_1_ signal is directly related to Mn^2+^ accumulation in an activity dependent manner, MEMRI allows the identification and quantification of neural activity. In the present study, T_1_ values obtained from MnCl_2_-infused rats were significantly lower in the cortex and thalamus of GBM rats fed ad libitum than controls under the same feeding condition. The decrease in T_1_ reflects a higher Mn^2+^ accumulation in the corresponding regions of GBM animals, thus revealing increased neuronal activation. These results are consistent with previous studies reporting abnormal neuronal excitability, and progressive desynchronization of neuronal activity, in contralateral brain regions of glioma-bearing mice [48]. In addition, several studies have shown that the infiltrative nature of GBM can induce neuronal hyperexcitability through an anomalous increase in the synaptic glutamate release and a decrease in GABAergic inhibitory signals [49, 50].

After 16 h of food deprivation, our MEMRI approach did not identify significant changes in T_1_, either in control or GBM fasted rats. Previous studies using MEMRI reported increased neuronal activity in the hypothalamic nuclei during a similar overnight fasting period in mice [25], or after 24 h of food deprivation, as well as in rats with dehydration-induced anorexia [24]. The first study, however, was methodologically different from the present one, as it involved a 2-h MnCl_2_ infusion performed simultaneously with MR acquisitions, instead of the current protocol which used a faster Mn^2+^ administration (approx. 25 min) 24 h prior to scanning. In addition, the increased signal in the arcuate nucleus (ARC) was observed to decay 1 h after the beginning of administration, revealing a relatively rapid diffusion process of Mn^2+^ out of the ARC into the remaining hypothalamic nuclei and potentially into other hunger-regulating structures. Furthermore, in both studies the authors imaged at higher magnetic fields (9.4 and 14.1 T) that allowed for sub-hypothalamic regionalization, which is less feasible in our 7 T system. Since the hypothalamic nuclei that comprise the hypothalamus are known to exhibit different activation patterns, it remains possible that our hypothalamic ROI averaged dissimilar responses, for example activation or inhibition, resulting in undetectable net changes.

Unexpectedly, the DTI studies performed 24 h after MnCl_2_ administration failed to detect any significant change in diffusion parameters with fasting. We have previously reported significant changes in brain diffusion coefficients in healthy mice, rats and humans after food deprivation, reflecting the orexigenic activity-induced cellular swelling responses to hunger [18, 35]. In the present study, we suspect that the Mn^2+^ administration interferes with the diffusion-detected brain response to starvation. Significantly lower FA values were found in the cortex, hippocampus and thalamus of fed tumor-bearing rats as compared to controls, and in the cortex and hippocampus of the same group under fasting. This suggests a loss of brain parenchymal integrity in animals with tumor, such as those detected in rat glioma models [51, 52] and in patients diagnosed with high-grade glioma [53, 54]. Thus, this decrease in the FA values in the GBM rats may involve a number of additional effects including increased cell density, lack of fiber integrity, cancer cell infiltration processes, and/or microvascular alterations Future research with microvascular and tractography dedicated MRI approaches and histological studies, will need to address the physiological origin of the FA changes reported here.

Mn^2+^ is known to trigger in the brain a neurotoxicity response [27], which involves neuro-inflammation [55]. Previous studies have also reported that Mn^2+^ can induce microglial activation by triggering the secretion of proinflammatory factors like TNFα, iNOS and IL-1β [56]. The release of these proinflammatory factors usually affects the integrity of the blood–brain barrier causing water extravasation and the appearance of vasogenic edema. Notably, an increase of the extracellular water content in the brain tissue is detected in diffusion MRI studies as an increase in the diffusion coefficients [10]. As mentioned above, according to previous investigations, food deprivation is expected to cause activity-induced cellular swelling, which is detected as a local decrease of MD in the regions involved. The present results do not show this finding, likely due to an opposing and overlapping neuroinflammatory effect. This result can derive from Mn^2+^ neurotoxicity that may be obscuring the normal brain response to fasting in Mn^2+^ infused animals. Although the timing of the manganese-related proinflammatory response is not clearly described our results suggest that this effect is likely to occur as soon as the cation accumulates in cells through the Ca^2+^ channels, and Mn^2+^ levels are known to increase significantly in the brain 24 h after administration [57].

To investigate whether the effects of Mn^2+^ actually interfered with the determination of water diffusion in the brain, we performed DTI studies in two new cohorts of rats not previously infused with MnCl_2_. Our data revealed that the control cohort showed a significant decrease in MD values after 16 h of food deprivation, as expected previously [18]. However, tumor-bearing rats presented comparable MD coefficients under fed and fasting conditions, suggesting that the normal brain response to fasting is altered in the GBM animals. Taken together, our data suggest that glioma induces important alterations in the brain, preventing a normal cerebral response to fasting, and probably contributing to the development of eating disorders as those described in brain cancer patients [5]. Indeed, abnormal cerebral responses and tumor-related neurocognitive dysfunction are widely reported circumstances in glioma patients [58–60], which may underlie loss of appetite in brain cancer patients, paving the way to cachexia [61].

Comparisons in non-Mn^2+^ infused between tumor and control cohorts under the same feeding conditions revealed significantly lower MD values in the cortex, hippocampus and hypothalamus of fed GBM rats, and in the hippocampus of fasted GBM animals. In this regard, increased cellularity in high-grade gliomas is reported to induce a decrease in water diffusion as the tumor grows [62, 63], and the return of the apparent diffusion coefficient to higher values with cancer treatment may be used as an early detection marker of the therapeutic success [62]. Thus, the decrease in MD observed in the contralateral region of rats with GBM may indicate long-range cell proliferation processes due to the infiltrative nature of the tumor, or even long-range compression effects derived from tumor growth. Both possibilities are well supported by changes in FA values, even in MnCl_2_-infused animals. Finally, pairwise fed vs. fasted comparisons in each independent cohort resulted in significantly fasting-reduced FA values in the hypothalamus of the control rats, and fasting-augmented FA values in the cortex of the tumor-bearing animals. Reduced FA values in the hypothalamic region of the control animals may represent the microstructural consequence of neurocellular swelling events associated with fasting. These swelling events physiologically resemble cytotoxic edema, a circumstance reported to be closely related to FA [64], although the molecular mechanisms remain to be elucidated. In GBM rats, the regional increases in FA probably again reflect compression effects arising from the presence of the tumor. As in the study with Mn^2+^ infused animals, FA values of non-Mn^2+^ infused animals were significantly lower in the cortex, hippocampus and thalamus of GBM rats under both fed and fasting conditions. This finding suggests once again that the decrease in FA in the contralateral hemisphere is likely related to the infiltrative nature of the tumor, the effects of compression, and the resulting microstructural changes.

Importantly, our study has several limitations. First, the number of animals per group may seem low. However, the total number of animals aligns with the bioethical considerations and a respectful use of laboratory animals. In addition, the sample size of the experimental groups in our work is very similar to those used in analogous preclinical studies with rats as models of brain pathologies. Second, the use of improved DTI sequences could allow us to segment nuclei in the hypothalamus and find alterations that were not seen in our current data. And third, histological studies could be performed to assess the brain alterations detected with DTI.

## Conclusion

The present study evaluated two complementary MRI techniques that provide functional information -DTI and MEMRI- in the assessment of brain responses to fasting in a glioblastoma model in rats. MEMRI studies identified increased neuronal excitability due to the presence of the tumor, although they could not detect the effects of overnight fasting. Moreover, our data revealed that the neurotoxic effects of Mn^2^ significantly interferes with the use of diffusion imaging methodologies. Although the molecular mechanisms underlying Mn^2+^ interference in diffusion-detected brain activation deserve further investigation, it is clear that these perturbances hinder the use of MEMRI and diffusion methodologies in the same MRI session.

In addition, DTI studies revealed microstructural alterations in the contralateral region of tumor-bearing animals. DTI in absence of Mn^2+^ did identify neurocellular swelling related to the fasting condition in healthy rats. However, in animals with advanced stage GBM, no changes were detected, suggesting impairment of hunger responses.

In summary, both MEMRI and DTI results suggest that tumor-induced changes in the brain alter the network necessary for proper regulation of energy balance, which provides further insight into the understanding of cancer-associated eating disorders and progression to cancer-associated anorexia and cachexia.

## Supplementary Information

**Additional file 1: Supplementary Figure 1.** Experimental design. **A** Animals included in the control and tumor-bearing cohorts, submitted to the infusion of a 100 mM MnCl_2_ solution 24 h prior the MRI session, that included MEMRI and DTI evaluations, under fed or fasted conditions. **B** Animals included in the control and tumor-bearing cohorts, in fed and fasted conditions, subjected only to DTI studies. The same animals were studied, one day apart, under the two-feeding status. **Supplementary Figure 2.** Representative images of an MRI session. The panels show the five slices acquired in a rat with GBM: **A **T_2_W images; **B** T_1_W images, from a MEMRI study, at TR = 400 ms; **C** diffusion images, from a DTI study, acquired in one direction at b = 200 s/mm^2^; **D** diffusion images from the same DTI study and direction, but acquired at b = 1000 s/mm^2^. The tumors are manually outlined with a white line. **Supplementary Table 1.** Mean value and standard deviation (SD) of T_1_ values 24h after MnCl_2_ infusion, for each cohort of rats, region, state and feeding condition. No statistical differences were found in the pairwise comparison of fed vs fasted animals for every brain region**. Supplementary Table 2.** Mean value and standard deviation (SD) of MD and FA, 24h after MnCl_2_ infusion, for each cohort, region, state and feeding condition. No statistical differences were found in the pairwise comparison of fed vs fasted animals for every brain region. **Supplementary Table 3.** Mean value and standard deviation (SD) of MD and FA of each cohort, region, state and feeding condition. *p*-values correspond to the comparison fed vs. fasted animals in the absence of Mn^2+^ infusion.

## Data Availability

The data presented in this study are available on request from the corresponding author due to the need for a formal data sharing agreement.

## Abbreviations

δ: Gradient diffusion duration
Δ: Gradient diffusion separation
ARC: Arcuate nucleus
Ca^2+^: Calcium ion
DTI: Diffusion tensor imaging
DWI: Diffusion weighted imaging
FA: Fractional anisotropy
GBM: Glioblastoma multiforme
GEE: Generalized estimating equations
GzLM: Generalized linear model
MD: Mean diffusivity
MEMRI: Manganese enhanced magnetic resonance imaging
Mn^2+^: Manganese ion
MRI: Magnetic resonance imaging
Mtx: Matrix data
ROIs: Regions of interest
TE: Echo time
TR: Repetition time
