# Supplementary material for: Magnetic resonance imaging to assess the brain response to fasting in glioblastoma-bearing rats as a model of cancer anorexia
Source: Cancer Imaging. 2023 Apr 10;23:36. doi: 10.1186/s40644-023-00553-y (PMC10088192; doi:10.1186/s40644-023-00553-y)
Supplement: Supplementary file 1 — Additional file 1: Supplementary Figure 1. Experimental design. A Animals included in the control and tumor-bearing cohorts, submitted to the infusion of a 100 mM MnCl2 solution 24 h prior the MRI session, that included MEMRI and DTI evaluations, under fed or fasted conditions. B Animals included in the control and tumor-bearing cohorts, in fed and fasted conditions, subjected only to DTI studies. The same animals were studied, one day apart, under the two-feeding status. Supplementary Figure 2. Representative images of an MRI session. The panels show the five slices acquired in a rat with GBM: A T2W images; B T1W images, from a MEMRI study, at TR = 400 ms; C diffusion images, from a DTI study, acquired in one direction at b = 200 s/mm2; D diffusion images from the same DTI study and direction, but acquired at b = 1000 s/mm2. The tumors are manually outlined with a white line. Supplementary Table 1. Mean value and standard deviation (SD) of T1 values 24h after MnCl2 infusion, for each cohort of rats, region, state and feeding condition. No statistical differences were found in the pairwise comparison of fed vs fasted animals for every brain region. Supplementary Table 2. Mean value and standard deviation (SD) of MD and FA, 24h after MnCl2 infusion, for each cohort, region, state and feeding condition. No statistical differences were found in the pairwise comparison of fed vs fasted animals for every brain region. Supplementary Table 3. Mean value and standard deviation (SD) of MD and FA of each cohort, region, state and feeding condition. p-values correspond to the comparison fed vs. fasted animals in the absence of Mn2+ infusion. [file 40644_2023_553_MOESM1_ESM.docx]

***Supplementary Material***

**Magnetic Resonance Imaging to Assess the Brain Response to Fasting in Glioblastoma-Bearing Rats as a Model of Cancer Anorexia**

Irene Guadilla^1^, Sara González^1^, Sebastián Cerdán^1^, Blanca Lizarbe^1,2^ and Pilar López-Larrubia^1^*

^1^Biomedical Magnetic Resonance Group, Instituto de Investigaciones Biomédicas Alberto Sols, CSIC-UAM, Madrid 28029, Spain

^2^Departamento de Bioquímica, Universidad Autónoma de Madrid, Madrid 28029, Spain

*** Correspondence:**

Pilar López-Larrubia

Instituto de Investigaciones Biomédicas Alberto Sols CSIC/UAM

c/ Arturo Duperier 4

Madrid 28029

Spain

Phone: +34 915 854 385

email: plopez@iib.uam.es

## *Supplementary Figures*


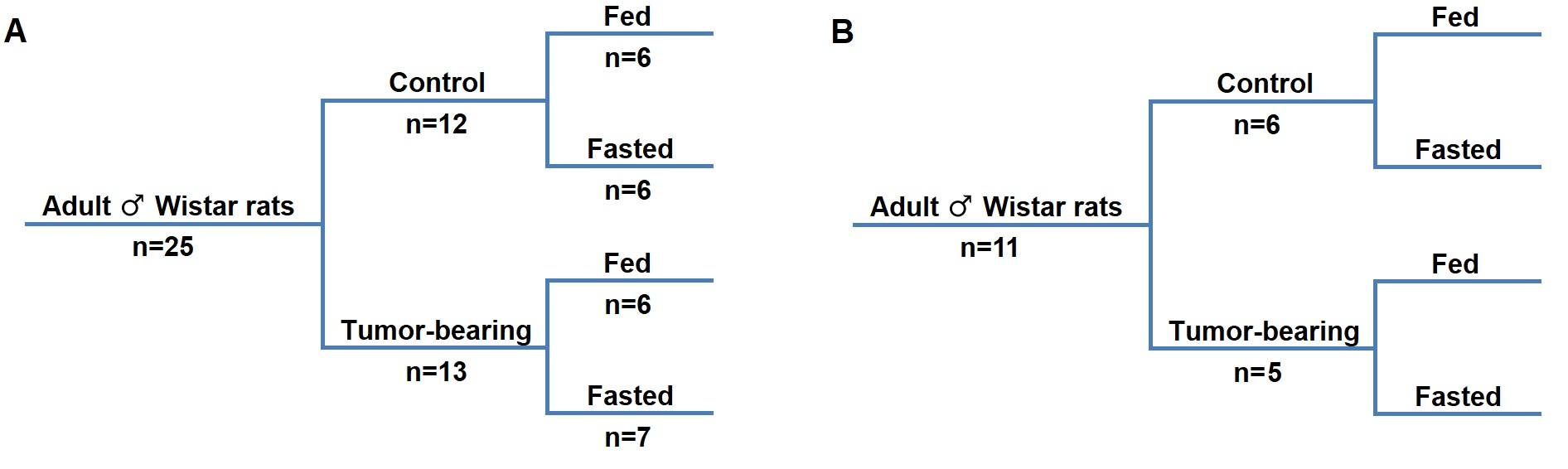


**Supplementary Figure 1.** Experimental design. **A** Animals included in the control and tumor-bearing cohorts, submitted to the infusion of a 100 mM MnCl_2_ solution 24 h prior the MRI session, that included MEMRI and DTI evaluations, under fed or fasted conditions. **B** Animals included in the control and tumor-bearing cohorts, in fed and fasted conditions, subjected only to DTI studies. The same animals were studied, one day apart, under the two-feeding status.


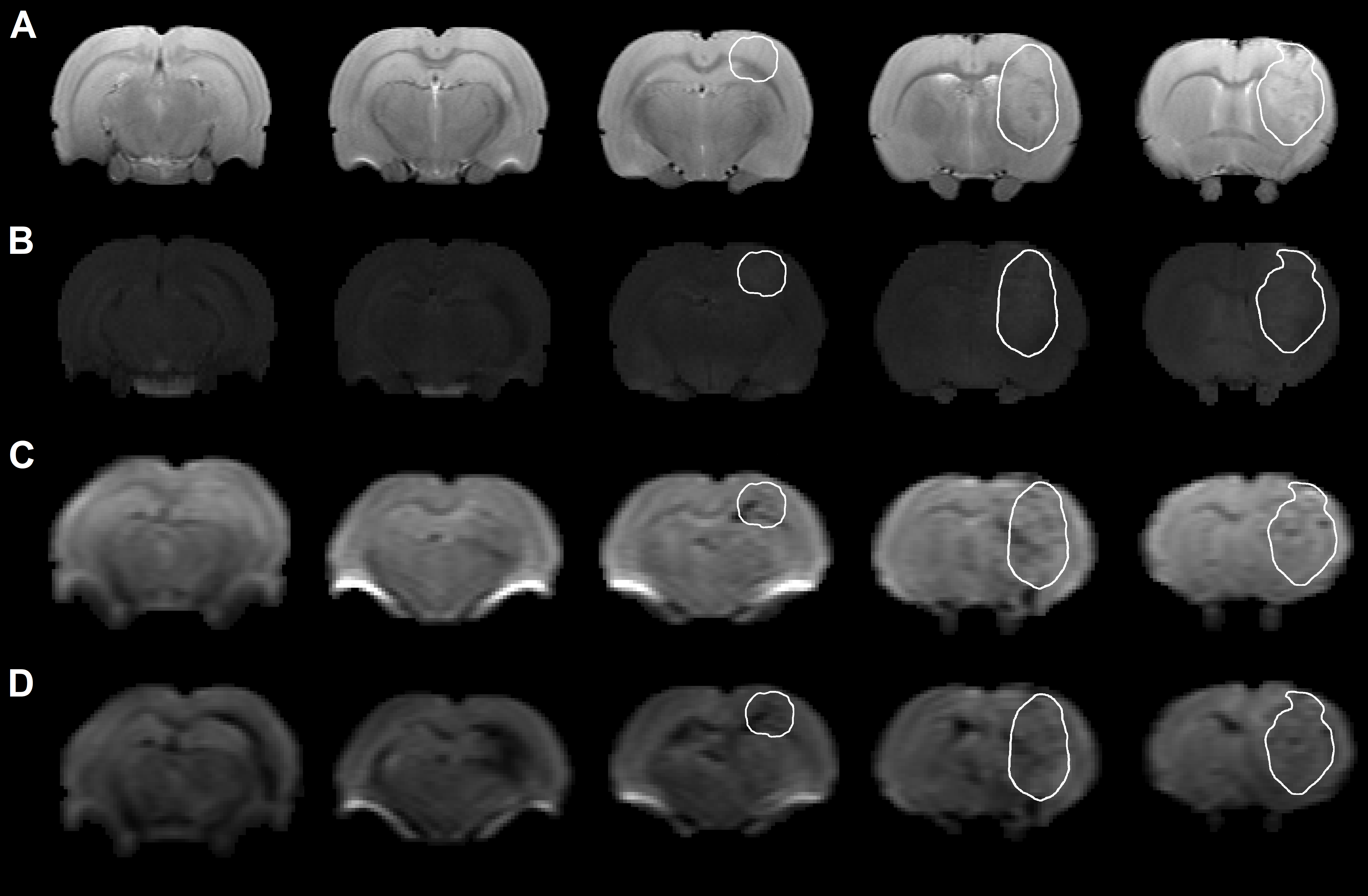


**Supplementary Figure 2.** Representative images of an MRI session. The panels show the five slices acquired in a rat with GBM: **A** T_2_W images; **B** T_1_W images, from a MEMRI study, at TR = 400 ms; **C** diffusion images, from a DTI study, acquired in one direction at b = 200 s/mm^2^; **D** diffusion images from the same DTI study and direction, but acquired at b = 1000 s/mm^2^. The tumors are manually outlined with a white line.

## *Supplementary Tables*

**Supplementary Table 1.** Mean value and standard deviation (SD) of T_1_ values 24h after MnCl_2_ infusion, for each cohort of rats, region, state and feeding condition. No statistical differences were found in the pairwise comparison of fed vs fasted animals for every brain region**.**

| Region | State | T1 (ms) Fed | | T1 (ms) Fasted | |
| --- | --- | --- | --- | --- | --- |
|  |  | Mean | SD | Mean | SD |
| Cortex | Control | 2392 | 142 | 2246 | 228 |
|  | Tumor-bearing | 2155 | 180 | 2358 | 358 |
| Hippocampus | Control | 2090 | 157 | 1997 | 177 |
|  | Tumor-bearing | 1968 | 220 | 2110 | 284 |
| Hypothalamus | Control | 1889. | 148 | 1816 | 188 |
|  | Tumor-bearing | 1924 | 209 | 1833 | 178 |
| Thalamus | Control | 2024 | 121 | 1926 | 146 |
|  | Tumor-bearing | 1893 | 166 | 1940 | 191 |

**Supplementary Table 2.** Mean value and standard deviation (SD) of MD and FA, 24h after MnCl_2_ infusion, for each cohort, region, state and feeding condition. No statistical differences were found in the pairwise comparison of fed vs fasted animals for every brain region.

| Parameter | Region | State | Fed | | Fasted | |
| --- | --- | --- | --- | --- | --- | --- |
|  |  |  | Mean | SD | Mean | SD |
| MD  (μm^2^/s) | Cortex | Control | 907 | 187 | 893 | 122 |
|  |  | Tumor-bearing | 791 | 58 | 818 | 66 |
|  | Hippocampus | Control | 960 | 184 | 973 | 142 |
|  |  | Tumor-bearing | 836 | 113 | 879 | 113 |
|  | Hypothalamus | Control | 947 | 199 | 909 | 132 |
|  |  | Tumor-bearing | 825 | 56 | 855 | 78 |
|  | Thalamus | Control | 871 | 127 | 909 | 122 |
|  |  | Tumor-bearing | 783 | 60 | 790 | 50 |
| FA | Cortex | Control | 0.41 | 0.07 | 0.40 | 0.11 |
|  |  | Tumor-bearing | 0.26 | 0.13 | 0.26 | 0.12 |
|  | Hippocampus | Control | 0.35 | 0.09 | 0.34 | 0.09 |
|  |  | Tumor-bearing | 0.20 | 0.10 | 0.23 | 0.13 |
|  | Hypothalamus | Control | 0.37 | 0.08 | 0.32 | 0.10 |
|  |  | Tumor-bearing | 0.32 | 0.11 | 0.29 | 0.10 |
|  | Thalamus | Control | 0.36 | 0.11 | 0.34 | 0.10 |
|  |  | Tumor-bearing | 0.20 | 0.09 | 0.27 | 0.11 |

**Supplementary Table 3.** Mean value and standard deviation (SD) of MD and FA of each cohort, region, state and feeding condition. *p*-values correspond to the comparison fed vs. fasted animals in the absence of Mn^2+^ infusion.

| Parameter | Region | State | Fed | | Fasted | | *p*-value |
| --- | --- | --- | --- | --- | --- | --- | --- |
|  |  |  | Mean | SD | Mean | SD |  |
| MD  (μm^2^/s) | Cortex | Control | 962 | 88 | 759 | 59 | **<0.001** |
|  |  | Tumor-bearing | 813 | 90 | 758 | 34 | > 0.05 |
|  | Hippocampus | Control | 1027 | 91 | 866 | 110 | **<0.001** |
|  |  | Tumor-bearing | 849 | 100 | 808 | 46 | > 0.05 |
|  | Hypothalamus | Control | 985 | 168 | 880 | 145 | > 0.05 |
|  |  | Tumor-bearing | 857 | 101 | 839 | 117 | > 0.05 |
|  | Thalamus | Control | 916 | 64 | 803 | 81 | **<0.001** |
|  |  | Tumor-bearing | 841 | 91 | 775 | 44 | > 0.05 |
| FA | Cortex | Control | 0.44 | 0.12 | 0.33 | 0.11 | > 0.05 |
|  |  | Tumor-bearing | 0.11 | 0.04 | 0.17 | 0.05 | **<0.001** |
|  | Hippocampus | Control | 0.40 | 0.12 | 0.30 | 0.10 | 0.057 |
|  |  | Tumor-bearing | 0.13 | 0.05 | 0.13 | 0.05 | > 0.05 |
|  | Hypothalamus | Control | 0.36 | 0.10 | 0.25 | 0.12 | **0.030** |
|  |  | Tumor-bearing | 0.23 | 0.16 | 0.18 | 0.05 | > 0.05 |
|  | Thalamus | Control | 0.37 | 0.14 | 0.30 | 0.11 | > 0.05 |
|  |  | Tumor-bearing | 0.14 | 0.07 | 0.15 | 0.04 | > 0.05 |
